# The Analysis of Arterial Stiffness in Heart Failure Patients in Comparison with Healthy Subjects and Patients with Cardiovascular Risk Factors

**DOI:** 10.3390/jcm8101721

**Published:** 2019-10-18

**Authors:** Mauro Feola, Marzia Testa, Cinzia Ferreri, GianLuca Rosso, Arianna Rossi, Gaetano Ruocco

**Affiliations:** 1 Ospedale Regina Montis Regalis, Cardiology Department, Mondovì, 12084 Cuneo, Italy; marzia.testa@aslcn1.it (M.T.); gaetanomaria.ruocco@aslcn1.it (G.R.); 2 University of Geriatrics, 10121 Turin, Italy; cinzia.ferreri@aslcn1.it (C.F.); arirossi@gmail.com (A.R.); 3 The 118 Emergency Department regione Piemonte, 10121 Turin, Italy; glrosso@hotmail.com

**Keywords:** blood pressure, arterial stiffness, heart failure, pulse wave analysis

## Abstract

The arterial stiffness in the pathogenesis and clinical outcome in heart failure (HF) patients still needs to be clarified. An increased pulse wave velocity (PWV) in HF patients in comparison with healthy subjects and cardiovascular risk factors (CVRF) patients has been demonstrated. The aim of this study was to evaluate the arterial stiffness in HF patients in comparison to control populations. Methods: Consecutive patients admitted for decompensated heart failure underwent echocardiogram and evaluation of arterial stiffness by measuring the PWV and the augmentation index (AIx75). The arterial stiffness was also calculated in a control group formed by healthy volunteers and in CVRF subjects. Results: Fifty-nine HF patients (62% males; age 75 years) with mean left ventricular ejection fraction (LVEF) 38% and N-terminal pro B-type natriuretic peptide (NT-proBNP) (8111 pg/mL) entered the study. The HF population were compared with 22 healthy controls (age 58 years) and 20 CVRF patients (age 72 years). The analysis of PWV demonstrated a velocity of 10.6 m/s (9–12.1 m/s), 11.7 m/second (10.4–12.8 m/s), and 10.1 m/second (8.6–10.8m/s) in controls, CVRF, and HF patients (*p* = 0.01). AIx75 was seen to be higher in the CVRF group vs. HF patients (34% vs. 22%, *p* = 0.001). In HF patients PWV was inversely correlated with the glomerular filtration rate (*r* = –0.40; *p* = 0.002) and directly with central systolic pressure (SP) (*r* = 0.29; *p* = 0.02), brachial SP (*r* = 0.33; *p* = 0.01) as well as AIx75 correlated with GFR (*r* = −033; *p* = 0.01). Conclusion: PWV proved to be different in HF patients in comparison with CVRF/healthy population. The strongest correlation was revealed between the values of PWV/AIx75 and renal function.

## 1. Introduction

Vascular ageing in large arteries plays an important role in contributing to cardiovascular morbidity and mortality [1]. Structural changes include an increase in wall thickness; the intima-media thickening ratio is widely attributed to sub-clinical atherosclerosis and it is independently correlated to cardiovascular prognosis [1].

The most marked functional change in large arteries with age consists in a real "stiffening". Arterial stiffness, assessed using pulse wave velocity (PWV), is also a strong independent predictor of cardiovascular events; [1] it plays an important role in terms of the age-related increase in systolic blood pressure and pulse pressure, which are both components of blood pressure closely associated with cardiovascular risk in middle-aged or elderly subjects [2].

The etiology of arterial stiffening should be related to degenerative/calcified processes; conversely, the thickening of the walls should be much more related to the atherosclerotic processes. However, it is not clear in which way both processes are correlated. Recently, the Consensus Document on the ventricular-arterial coupling in cardiac disease, recognized PWV as the gold standard non-invasive examination capable of studying large artery stiffness. Furthermore, the document explored the significance of arterial stiffness in heart failure with reduced ejection fraction (HFrEF) and heart failure with preserved ejection fraction (HFpEF), considering the analysis of the ventricular-arterial coupling to be extremely useful in managing therapy [3].

In our study, comparing three different groups (healthy subjects, subjects with cardiovascular risk factors, and patients suffering from heart failure) we aimed: a) To observe whether the degree of arterial stiffness, measured by a non-invasive method, proved to be different in heart failure (HF) patients in comparison with controls; and b) to evaluate if in heart failure (HF) patients a correlation between arterial stiffness and cardiac function or plasma biomarkers emerged.

## 2. Methods

The present study is an observational study performed from June 2018 to January 2019, at the Cardiological Rehabilitation/Failure Unit of the SS Trinity Hospital in Fossano (CN, Italy) and the Cardiology Department of the Regina Montis Regalis Hospital in Mondovì (CN, Italy). Hospitalized patients aged ≥65 years following acute heart failure were consecutively enrolled; all patients gave their written informed consent. Our inclusion and exclusion criteria are described below.

### 2.1. Inclusion Criteria

- Subjects consecutively admitted to the Cardiac Rehabilitation/Heart Failure Unit of the SS Trinità Hospital, Fossano, or the Cardiology Department of the Regina Montis Regalis Hospital, Mondovì, for acute heart failure; the clinical diagnosis of HF is entrusted to the Framingham criteria [4];
- The presence of 2 major criteria (paroxysmal nocturnal dyspnoea and orthopnea, jugular turgor, crackling rales, cardiomegaly, III tone, hepato-jugular reflux) or 1 major criterion and 2 minor criteria (ankle edema, dyspnea on exertion, hepatomegaly, nocturnal cough, pleural effusion, tachycardia); age ≥65 years.

### 2.2. Exclusion Criteria

- Impossibility in determining aortic stiffness;
- Absence of therapeutic compliance.

### 2.3. Baseline Evaluation

In all patients enrolled, a baseline evaluation was performed, through the compilation of a standardized evaluation form (CRF), which included:

1. Demographic parameters (date of birth, age, sex);
2. Accurate remote pathological anamnesis with careful evaluation of traditional cardiovascular risk factors (diabetes mellitus, type of cardiomyopathy, New York Heart Association [NYHA] class);
3. Blood chemistry tests including hemoglobin, sodium, creatinine, estimated glomerular filtration rate (eGFR) using the Cockcroft-Gault formula, and measurement of N-terminal pro B-type natriuretic peptide (NT-proBNP) levels;
4. Transthoracic echocardiogram.

In addition, in this study we included two control groups formed by: A healthy group (composed of young and healthy subjects) and a cardiovascular risk factor group, which included subjects with at least one cardiovascular risk factor (such as smoking, hypertension, diabetes mellitus type 2, and dyslipidemia) but in a stable hemodynamic condition. All subjects in the control groups underwent a cardiological examination and a 12-lead ECG in order to exclude the presence of a heart failure condition.

**NT-proBNP** is a rapid immunochromatographic assay on serum, plasma, and whole blood. The specific antibodies are immobilized on the nitrocellulose membrane in the T (test) line of the device. The test results must be interpreted within 10 minutes. A normal value for the test is considered <250 ng/L.

**Echocardiograms** were performed with a GE Vivid 7 Pro, according to the recommendations of the American Society of Echocardiography [5]. Two-dimensional apical 2- and 4-chamber views were used for volumetric measurements; left ventricular ejection fraction (LVEF) was calculated with a modified Simpson’s method using biplane apical (2- and 4-chamber) views. The LV end-diastolic volume and end-systolic volumes were recorded. All the echo examinations were performed by expert operators blinded to the results of BNP assay; the intra-observer variability in the evaluation of LVEF was <5%. Echocardiographic measurements included LV end-diastolic diameter and diastolic thickness of the ventricular septum and posterior LV wall. Left ventricular systolic dysfunction was defined as an LVEF < 50%. Diastolic dysfunction was graded according to: 

(1) **Impaired relaxation**: Early filling wave (E)/atrial filling wave (A) ratio (E/A) < 1 and deceleration time (DT) > 220 msec in subjects aged <55 years or E/A < 0.8 and DT > 220 msec in subjects aged >55 years; systolic wave (S)/diastolic wave (D) ratio of pulmonary veins (PV) (PV S/D) > 1, and atrial reversal flow velocity (AR) < 35 cm/second;

(2) **Pseudonormal**: E/A 1 to 2, DT 150 to 220 msec, PV S/D ratio < 1, and AR > 35 cm/second;

(3) **Restrictive**: (E/A > 2, DT < 150 msec, S/D ratio < 1, and AR > 35 cm/second).

In patients suffering from atrial fibrillation at the time of the echocardiogram, the diastolic function was classified as: 1) Restrictive pattern (DT < 150 msec) or 2) indeterminate (DT > 150 msec). The patients were also classified for left ventricular dilatation (end-diastolic diameter >57 mm). The pulmonary artery pressure (PAP) was obtained by determining the peak velocity of the tricuspid regurgitation jet, plus 5 or 10 mmHg for right atrial pressure according to right atrial size, severity of regurgitation, and appearance of the inferior vena cava. From Doppler tissue imaging of the annulus, the E’ wave (early annular velocity opposites in direction to the mitral inflow) was determined and the ratio E/E’ calculated. Right ventricular function was investigated by M-mode echocardiography, obtaining the tricuspid annular plane systolic excursion (TAPSE). The medical therapy was normally taken and the BNP measurements were obtained before the echo examination.

### 2.4. PWA and PWV Measurement: Central Blood Pressure Measurement and Pulse Velocity

In the study population, arterial stiffness parameters were evaluated using SphygmoCor XCEL (AtCor Medical, Itasca, IL, US), a non-invasive diagnostic tool for the clinical evaluation of central arterial pressure. The SphygmoCor XCEL System derives the central wave-shaped aortic pressure from the pulsations of the brachial artery cuffs. Waveform analysis provides key parameters that include central systolic pressure, central pulsation pressure, and arterial stiffness indices such as increased pressure and increase index. The increase in central systolic blood pressure and the increase indexes (Augmentation index) have been reported as indicators of cardiovascular risk.

The SphygmoCor XCEL System also measures the wave velocity of the pulse waveform of the arterial pulse moving from the descending aorta to the femoral artery (PWV). The velocity of the arterial pulse wave is detected by the carotid and femoral arterial impulses simultaneously measured in a non-invasive manner. The carotid pulse is measured through the tonometer while the femoral pulse is measured through the pulsations with a cuff placed around the thigh. PWV values in normal ranges depend on the age of the examined subjects, but can be considered within 9–10 m/second; obviously, an increase in the wave velocity of the carotid and femoral impulses indicates an increase in aortic stiffness, or damage to the target organ. Measurements were performed on a supine patient, in a quiet environment, excluding smoking in the hour before the examination or having abused vasoactive substances (coffee), keeping intact its pharmacological therapy. The Augmentation Index (AIx75) was measured at the level of the carotid artery by obtaining ten high quality pulse wave measurements with automatic calculation of AIx using the manufacturer’s proprietary software and after normalizing to a heart rate of 75 beats per minute, and represents the pressure boost that is induced by the return of the reflected waves at the aorta.

### 2.5. Statistical Analysis

Analysis of normal distribution of out variables was performed with Kolgomorov Smirnov test. Because of our parameters were not normally distributed, continuous variables were expressed as median (inter-quartile-range [IQR]). Qualitative variables were expressed as count (n.) or percentage (%). Differences among our subgroups of patients were calculated by non parametric tests such as Mann–Whitney test for two groups and Kruskal–Wallis test for three groups and were expressed as median. The differences between qualitative/categorical variables were analyzed by the chi-square test. Due to abnormal distribution of our variables, analysis of correlation was performed with non-parametric tests such as Spearman rho’s correlation coefficient. We considered results associated with a *p* ≤ 0.05 to be statistically significant. We used the SPSS software (version 20.0, Chicago, IL, USA) for all analyses.

## 3. Results

A total of 101 subjects were included in this study. Our subjects showed a mean age of 68 ± 13.9 years, 62% of whom were males, divided into a healthy group (22 subjects) and a cardiovascular risk factor group (CVRF, 20 subjects), both of which were evaluated by a cardiologist and considered suitable for non-competitive sports; a third group, called the HF group, was characterized by hospitalized patients due to de novo acute heart failure (AHF) or an exacerbation of chronic heart failure (CHF). Detection of aortic stiffness in decompensated patients was achieved in pre-discharge, clinical stability. The healthy group was represented by 22 subjects with a slightly lower median age than the other two groups (58 (40–66) years), whereas the CVRF group included 20 patients with a higher median age of 72 (60–77) years. In the CVRF group, 2 (10%) were diabetics, 18 had hypertension (90%), and 6 (33.3%) were active smokers.

The HF group, on the other hand, included 59 patients with a median age of 75 (70–81) years, of whom 33 patients (55.9%) demonstrated an ischemic etiology of HF (in eight cases treated with surgical myocardial revascularization and nine with percutaneous revascularization); six patients (10.1%) were affected by a valvular heart disease, 20% had dilatative cardiomyopathy, 14% hypertensive cardiomyopathy; 10 patients (17%) were diabetic. Just over half of patients (53%) were in sinus rhythm and seven patients (11.9%) were treated by the implantation, in primary prevention, of an implantable defibrillator (ICD). Table 1 shows the main descriptive and blood chemistry features of the examined sample. Median left ventricular ejection fraction (LVEF) of the HF group was 38% (30–45%); 13 patients (22%) showed HFpEF (defined as LVEF ≥ 50%), 30 patients (50.8%) showed HFrEF (defined as LVEF < 40%), and 16 patients (27.2%) showed heart failure with mid-range ejection fraction (HFmrEF, LVEF 40%–49%). During admission for acute heart failure (AHF), a significant neuro-hormonal activation occurred [NT-proBNP values of 8111 (3258–20,180) ng/L]. The NYHA class at admission was found to be III–IV on average, but had improved at discharge (NYHA class II in 39%, class III in the remaining cases). In the HF population a mild renal impairment (creatinine 1.01mg/dl; range 0.82–1.79) was demonstrated. The in-hospital stay was about 10 days (9.9 ± 4.4 days). At discharge, 98% of patients were treated with loop diuretics, 91.6% beta-blockers, 36.1% assumed ACE-inhibitors/ARBs, 66.6% antialdosteronic drugs, 13.5% ivabradine, and, finally, 13.5% sacubitril/valsartan. One patient (1.7%) died during hospitalization due to multi-organ failure.

**Figure 1:**
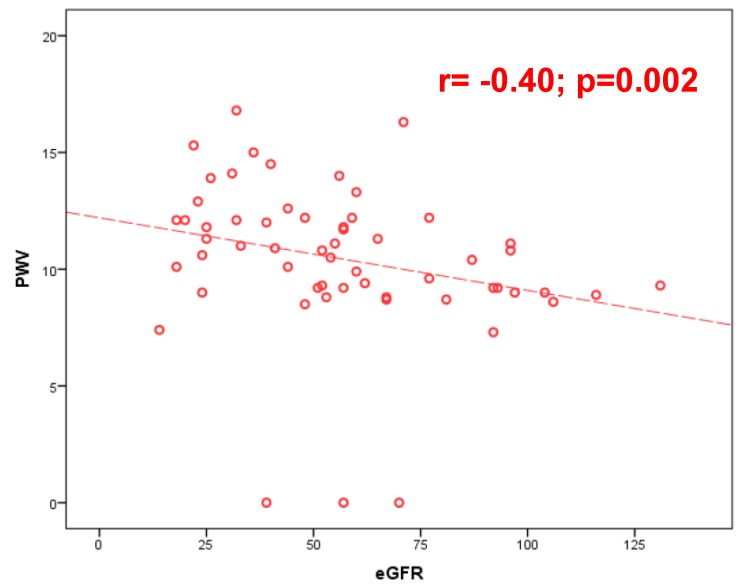
Spearman’s Rho correlation coefficient between pulse wave velocity (PWV) and estimated glomerular filtration rate (eGFR).

**Figure 2:**
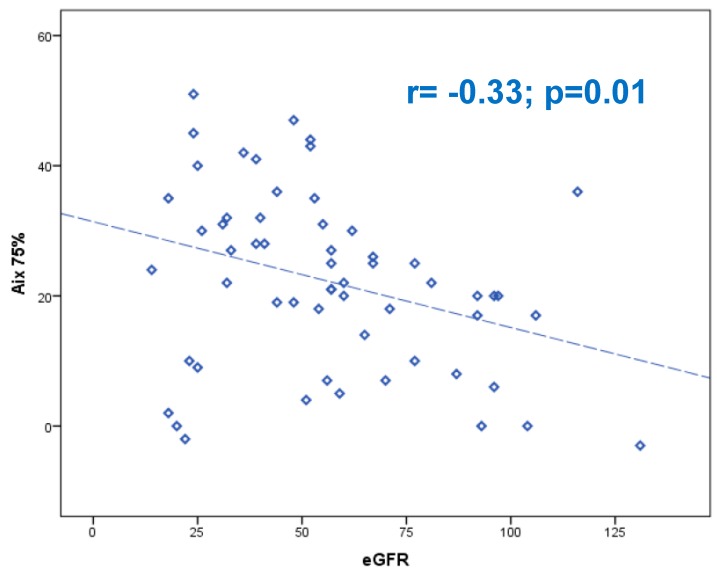
Spearman’s Rho correlation coefficient between the augmentation index at 75 bpm (AIx75) and eGFR.

By analyzing the entire population, we showed that PWV was significantly correlated with brachial systolic pressure (BSP) (*r* = 0,49; *p* < 0,001), central systolic pressure (CSP) (*r* = 0,46; *p* < 0.001), brachial pulsatory pressure (BPP) (*r* = 0.36; *p* < 0,001), and central pulsatory pressure (CPP) (*r* = 0,29; *p* < 0,001). In the HF group (59 patients), PWV demonstrated a positive moderately significant correlation with creatinine (*r* = 0.33; *p* = 0.01), RDW (*r* = 0.31; *p* = 0.02); NT-proBNP (*r* = 0.28; *p* = 0.049), brachial SP (*r* = 0.33; *p* = 0.01), central SP (*r* = 0.29; *p* = 0.02), and a negative moderately significant correlation with eGFR (*r* = −0.40; *p* = 0.002) (Figure 1). The AIx75 showed a positive, weakly significant correlation with creatinine (*r* = 0.27; *p* = 0.04), sodium (*r* = 0.28; *p* = 0.04), central PP (*r* = 0.43; *p* = 0.001), and a negative moderately significant correlation with eGFR (*r* = −0.33; *p* = 0.01) (Figure 2). Dividing our population according to the LVEF (HFrEF = 30/59 pts; mid-range HF = 16/59 pts, and HFpEF = 13/59 pts), the median of PWV (10.8 m/s; 10.1 m/s; 10.5 m/s, respectively) and the AIx75 (21%, 24.5%, 25%, respectively) did not change significantly (*p* = 0.7 and *p* = 0.6) between subgroups. The analysis of PWV and AIx75 divided for left ventricular diastolic function (0 = normal, type 1-2-3) did not show significant differences (*p* = 0.45 and *p* = 0.73, respectively) either. Finally, Table 2 describes the differences of age and arterial stiffness parameters in the three subgroups. 

## 4. Discussion

Nowadays, the role of the aortic stiffness values in HF patients is under debate. Moreover, the prognostic meaning of a differing degree of PWV/AIx75 in these patients has not been elucidated yet. Weber and Chirinos [6] recently highlighted in their clinical review that central pressure and wave reflections are both related to the left ventricular late systolic afterload, ventricular remodeling, diastolic dysfunction, and the risk of new-onset HF. [6] In fact, left ventricular hypertrophy, a marker of organ damage in hypertension, is an important intermediate step from hypertension to HF [7]. Left ventricular mass seemed to be more correlated to PP than to mean arterial pressure, confirming the importance of the pulsatile phenomena and the measurement of it [8]. In advanced HFrEF patients, a low brachial PP is due to a poor left ventricle function and has been associated with a worse prognosis. With a less severe degree of HRrEF, PP seemed to be more reflective of arterial stiffness, increased pulsatile afterload worsening the hemodynamic conditions. In acute decompensation patients, Sung et al [9] demonstrated the adverse prognostic value of wave reflections in 80 acute HF patients in a short-term follow-up (six months) [9]. Regnault et al., in the EPHESUS study, studying 306 post-MI HF patients with systolic dysfunction (LVEF < 40%) demonstrated that a higher PP correlated with a lower events-rate and that an increased PWV was associated with a negative prognosis [10]. Similarly, the measurement of PWV as an expression of arterial stiffness has been associated with an increment of HF hospitalization and CV mortality in chronic, stable HFpEF [11]. Nagele et al. documented in 74 stable HF patients a significant increase in PWV in comparison to healthy controls and CVRF patients, not evidencing, however, any difference in AIx75 between groups (*p* = 0.51 and *p* = 0.9, respectively) [12]. On the other hand, in 50 HF patients with advanced systolic dysfunction (LVEF < 30%), Abolfazl et al. did not observe any statistically significant correlation with adverse prognosis in a short-term follow-up (six months) of PWV underlying as invasive cardiac output PP and age were independent predictors of the composite endpoints [13].

Our clinical experience deserves three main considerations. 

(a) PWV proved to be different in HF patients in comparison with CVRF/healthy population. This result seemed to reinforce the possible role of PWV in HF development, even if an enlargement of the experience is necessary to drive a more significant conclusion. Unfortunately, the group of healthy subjects were younger in comparison with CVRF and HF patients; however, it seems very difficult to include healthy elderly subjects without any cardiovascular risk factors. Moreover, with similar age, CVRF subjects had higher brachial SP and central SP than HF patients, which might influence the result obtained for PWV, confirming the strict dependence of PWV on central/brachial BP. PP is a parameter determined by cardiac function and arterial stiffness through wave reflections. Large-artery stiffness, influenced by ageing, diabetes mellitus, atherosclerosis, and renal failure, is the main determinant of PP. In our clinical experience, central/brachial PP was not different in the three populations, confirming the results of Regnault et al. in which PP being negatively associated with prognosis, should not be considered a marker of aortic elasticity for its dependence to left ventricular function [12]. The limit number of HF patients examined did not permit a comparison between different types of HF (ischemic, idiopathic, etc.) in the analysis of different parameters of arterial stiffness, as well as the role in the prognosis of those patients. 

(b) The strongest correlation was revealed between the values of PWV/AIx75 and the value of renal filtrate (GFR). This data confirms that the presence of renal failure plays a role in the development of both heart failure and vascular damage even with an increase in aortic stiffness. Renal failure and ageing are two of the main determinants of the arteriosclerosis characterized by direct structural changes including elastin fragmentation and medial calcification that increased the arterial stiffness [14]. In renal failure, indeed, a combination of active processes adding a reduction in calcification inhibitors occurred, modifying calcium and phosphate metabolism and determined a calcification of intima and media of the vessel wall [14]. The 2018 European Society of Cardiology Guidelines underlined that a threshold of 10 m/s for PWV was reported as clinically correlated to an increased cardiovascular risk [15]. Data coming from a systemic meta-analysis, reported as the relative risk for all-cause mortality was found to be 1.15 for an increase in 1 m/s in PWV [16]. In renal failure patients, evaluation of PWV in 150 non-dialysis renal disease patients, demonstrated an early onset of elevated aortic stiffness and increased rate of progression over a year in those patients in comparison to non-renal disease patients [17].

(c) Finally, a significant difference in the values of AIx75 between the group of HF patients vs. CVRF and healthy group emerged (22% vs. 34% and 32%, respectively), being significantly reduced in the decompensated patients. A possible explanation should be sought in the formula that determines the Augmentation Index (Augmentation Pressure/Pulse Pressure). According to this formula, we deduce that a lower ratio is determined by the variations in the numerator or denominator value. Since the denominator (Pulse Pressure) is similar in the two groups (55 mmHg in the HF group, 60 mmHg in the CVRF group), it can be hypothesized that what decreased was the Augmentation Pressure in the HF patients. This data allows for the hypothesis that the reduction/delay of the arrival of the reflected wave could enter into the determinism of the left ventricular remodeling, since the arrival of the reflected wave creates an additional obstacle to the ejection of the left ventricle. Indeed, as elucidated by Weber and Chirinos, the dysfunctional ventricles, having a concentric remodeling/systolic dysfunction, fail to protect the myocardiocytes from the increase in work given by the delay of the reflection wave, which falls into a period of vulnerability due to the increase in load. [6] This might represent a vicious circle that favors the development and progression of heart failure. In addition, considering our results, in the HF patients more than the PWV, the AIx75 should be measured in order to evaluate the systolic delay of the wave reflections.

However, in this study, these measurements (e.g., PWV and AIx75) were performed during hospitalization but after acute decompensation. This choice is related to the methodology for data collection. Indeed, patients may be supine and may have a heart rate of 75 beats per minute. In the first phases of acute decompensation, it is not possible to collect these parameters correctly. Moreover, this is a pilot study and our results encouraged us to begin follow-up data collection to evaluate prognostic weight of both PWV and AIx75 in HF. 

## 5. Conclusions

In conclusion, it can be affirmed that in HF patients the low value of AIx75 could be considered in the determinism of heart failure. Brachial/central PP should not be considered a marker of aortic elasticity in HF patients. In the search for a prognostic significance in HF patients, aortic stiffness seems to be particularly related to renal dysfunction, which is extremely important in the prognostic setting. Further follow-up studies are mandatory to confirm prognostic power of these parameters in clinical practice. 

## Figures and Tables

**Table 1:** General characteristics of the heart failure population.

Population	59
Male Sex	62%
Female Sex	38%
Median Age	75 (70–81)
HF etiology	
Ischemic CMP	55.9%
Hypertensive CMP	14%
Dilated CMP	20%
Valvular CMP	10.1%
Diabetes	17%
Known CHF	25%
ICD	7%
LVEF (%)	38 (30–45)
NT-proBNP (pg/mL)	8111 (3258–20,180)
Sodium (meq/l)	140 (137–142)
Creatinine (mg/mL)	1.09 (0.82–1.79)
GFR (mL/min/1.73mq)	54 (33–72)
Hemoglobin (g/dL)	12.5 (11.2–14)
LVEDD (mm)	62 (52–65)
LVESD (mm)	46 (37–58)
TAPSE (mm)	19 (16–20)
PAP (mmHg)	40 (29–50)

CMP: Cardiomyopathy; VALVULAR: Valvular heart disease; ICD: Implantable cardioverter-defibrillator; LVEF: Left ventricular systolic function; NT-proBNP: NT-pro natriuretic peptide; GFR = glomerular filtration rate; LVEDD = left ventricular diastolic diameter; LVESD = left ventricular systolic diameter; TAPSE = tricuspid annular plane systolic excursion; PAP = pulmonary artery pressure.

**Table 2:** The results of arterial stiffness determination and age in the heart failure (HF) group and in the two control groups.

	HF Group (59 Patients)	CVRF Group (20 Subjects)	Healthy Group (22 Subjects)	*P*	*p*
PWV (m/sec)	10.6 (9–12.1)	11.7 (10.4–12.8)	10.1(8.6–10.8)	0.01	0.009
AIx75	22 (10–32)	34 (29–44)	32 (22–45)	0.001	0.001
BrachialSP (mmHg)	122 (111–132)	134 (126–144)	124 (112–132)	0.03	0.03
BrachialPP (mmHg)	53 (43–65)	60 (48–72)	51 (45–58)	0.15	0.15
CentralSP (mmHg)	111 (100–121)	125 (113–135)	114 (103–123)	0.005	0.005
CentralPP (mmHg)	39 (32–50)	50 (36–60)	41 (32–47)	0.10	0.10
Age (years)	75 (70–81)	72 (60–77)	58 (46–66)	0.001	

PWV = pulse wave velocity; AIx75 = augmentation index at 75 bpm; SP = systolic pressure; PP = pulsatory pressure.
